# Naturally Inspired Molecules as Multifunctional Agents for Alzheimer’s Disease Treatment

**DOI:** 10.3390/molecules21050643

**Published:** 2016-05-16

**Authors:** Angela Rampa, Andrea Tarozzi, Francesca Mancini, Letizia Pruccoli, Rita Maria Concetta Di Martino, Silvia Gobbi, Alessandra Bisi, Angela De Simone, Francesco Palomba, Nelsi Zaccheroni, Federica Belluti

**Affiliations:** 1 Department of Pharmacy and Biotechnology, Alma Mater Studiorum—University of Bologna, Via Belmeloro 6, 40126 Bologna, Italy; francymancini@gmail.com (F.M.); ritamaria.dimartino2@unibo.it (R.M.C.D.M.); silvia.gobbi@unibo.it (S.G.); alessandra.bisi@unibo.it (A.B.); 2 Department for Life Quality Studies, Alma Mater Studiorum—University of Bologna, Corso D’Augusto 237, 47921 Rimini, Italy; andrea.tarozzi@unibo.it (A.T.); letizia.pruccoli@studio.unibo.it (L.P.); angela.desimone2@unibo.it (A.D.S.); 3 Department of Chemistry ‘‘G. Ciamician’’, Alma Mater Studiorum—University of Bologna, Via Selmi 2, 40126 Bologna, Italy; francesco.palomba3@unibo.it (F.P.); nelsi.zaccheroni@unibo.it (N.Z.)

**Keywords:** AD, BACE-1, benzophenone, chalcone, metal chelation, natural products, Reactive oxygen species

## Abstract

Alzheimer’s disease (AD) has been defined as a multi-factorial disorder resulting from a complex array of networked cellular and molecular mechanisms. In particular, elevated levels of Aβ protein and its aggregation products in the presence of metal ions proved to be highly neurotoxic and therapeutic strategies aimed at preventing Aβ generation and oxidative stress may represent an effective approach for AD treatment. A recent paradigm for the treatment of complex diseases such as AD suggests the employment of multifunctional compounds, single chemical entities capable of simultaneously modulating different targets involved in the pathology. In this paper, the “pharmacophores combination” strategy was applied, connecting the main scaffold of the BACE-1 ligand **1** to that of the chalcone **2**, as metal chelating pharmacophore, to obtain a small library of compounds. Conjugate **5** emerged as the most interesting derivative, proving to inhibit BACE-1 with low-micromolar potency, and showing neuroprotective effects. In particular, **5** proved to be able to protect from metal-associated oxidative stress by hampering intracellular Cu^2+^-induced ROS formation without any direct neurotoxic effect.

## 1. Introduction

Alzheimer’s disease (AD), characterized by a progressive decline of cognitive and behavioral functions, represents a considerable social, economic, and health burden due to its ever-increasing incidence and prevalence. This is currently estimated to affect 24 million patients and this number is predicted to increase fourfold by the year 2050 [1]. AD has been defined as a multi-factorial disorder, being the result of a complex array of networked cellular and molecular mechanisms contributing to neuronal dysfunction and degeneration. A recent paradigm to treat complex diseases, widely accepted by the medicinal chemistry community, suggests the use of multifunctional compounds (MFCs), also called multitarget drugs, which are single chemical entities capable of simultaneously modulating different targets or pathways involved in the pathology [2,3].

A number of key pathological changes can be observed in AD brain, namely amyloid-β (Aβ) peptide extracellular deposits (amyloid neuritic plaques) and intracellular hyperphosphorylated tau protein-based microtubule assemblies (neurofibrillary tangles). Additional features include membrane-associated oxidative stress, neuroinflammation, biometal dyshomeostasis, mitochondrial dysfunction and extensive loss of neurons and synapses. To date, the exact mechanisms leading to these alterations still have to be fully uncovered.

A large body of evidence suggests that elevated levels of Aβ protein and its aggregation products, such as pre-fibrillary small soluble Aβ oligomers and dimers, are highly toxic species. They positively correlate with disease severity [4] due to their ability to trigger a cascade of neurobiological events, namely oxidative damage, neuroinflammation and neurotoxicity, ultimately culminating in brain atrophy, synaptic dysfunction and neuronal cell death [5]. Consequently, therapeutic strategies aimed at preventing Aβ generation and toxicity may represent an effective approach for AD treatment. Aβ protein is the results of amyloid precursor protein (APP) sequential cleavage by β- and γ-secretases. In particular, β-secretase (also known as β-site APP cleaving enzyme-1, or BACE-1), the aspartyl protease involved in the catalysis of the initial and rate-limiting step of APP processing, has been validated to play a key role in regulating the formation of Aβ and related post-translational products. Clinical trials outcomes demonstrated a direct correlation between BACE-1 inhibition and Aβ reduction. Moreover, the elevated BACE-1 protein expression detected in AD patients brains [6] elected this enzyme as attractive molecular target for disease-modifying drug discovery programs focused on the development of “anti-amyloid” therapeutics. Substantial efforts have been put into the discovery of small molecules as BACE-1 inhibitors in both academia and industry [7]. The various X-ray crystal structures of BACE-1 alone and in complex with an inhibitor represent an essential source of structural information for structure-based development of non-peptidic BACE-1 inhibitors with different core templates [7,8,9].

Several metal ions, among which Cu(II), Fe(II) and Zn(II), largely found in AD plaques, are known to be responsible for oxidative stress and neuronal cell death by promoting Aβ assembly and enhancing reactive oxygen species (ROS) production [10]. Chemical entities endowed with metal chelation properties proved to be able to hamper ROS generation and toxicity, together with metal-Aβ interaction, and could represent a valuable approach for reducing neurotoxicity [11].

Natural products (NPs), small molecules synthesized by the plant kingdom, can be defined “privileged structures” as they have undergone the natural selection process that allowed them to achieve structural (chemical) features for optimal interactions with a wide range of biological targets. Consequently, NPs possess different biological activities, responsible for a combination of therapeutic effects. During the last decades, NPs represented an excellent source of lead compounds [12] and this encouraged their employment as biologically pre-validated starting points for NPs-based approaches of drug design and discovery [13]. In this context flavonoids, a class of polyphenolic NPs, showed a wide therapeutic potential due to their ability to treat several human diseases including cancer, inflammation and AD. They can be regarded as multitarget compounds, since various molecular mechanisms are responsible for a single pharmacological effect. NPs proved to exert neuroprotective activity by affecting different AD networked pathways and acting through different mechanisms of actions, including suppression of neuroinflammatory responses and protection from neurotoxic injuries, such as those derived from Aβ species, metal ions and oxidative stress [14]. Their metal chelation ability was shown to depend on the presence of well-defined chelation sites in their structures, such as a carbonyl function adjacent to a hydroxy group. The flavonoid-based metal chelates showed an enhancement of several beneficial biological activities, among which the antioxidant effects, compared to the corresponding simple flavonoids [15].

### Design Strategy

**Figure 1:**
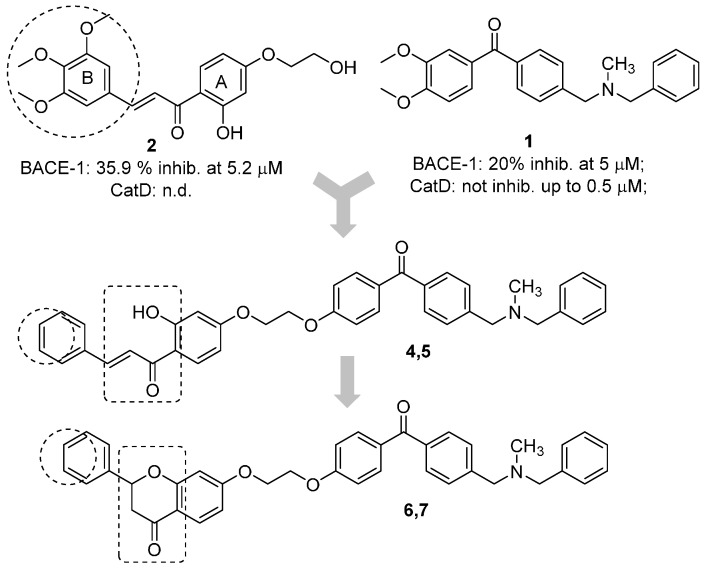
Design strategy for the optimization of the naturally inspired hits (**1** and **2**) to obtain conjugates (**4**–**7**) as BACE-1 inhibitors. Inhibitory activities of **1** and **2** are reported.

The benzophenone core is known to be a privileged structure, suitable to be exploited to provide effective ligands for different molecular targets involved in AD [16,17]. In an effort aimed at obtaining multipotent AD therapeutics, our in-house collection of naturally inspired small molecules was screened against the BACE-1 enzyme, and some promising hit compounds were identified. Among them, compounds **1** and **2** (Figure 1), bearing a benzophenone and a chalcone framework, respectively, showed a promising BACE-1 inhibition (36% and 20%, respectively) when tested at 5 µM concentration. In a previous work, the benzophenone **1**, bearing a *N*,*N*’-benzylmethylamine moiety, was selected as the main scaffold to be optimized, allowing us to obtain a mutitarget AD-drug candidate able to efficiently inhibit BACE-1 enzyme (low-micromolar potency) and to offer protection against Aβ-induced neurotoxicity as well [18]. Moreover, computational studies showed that the *N*,*N*’-benzylmethylamine function of **1** could appropriately interact with the catalytic dyad of the enzyme. As for the second selected scaffold, chalcones (1,3-diarylpropen-1-ones) occupy a special place as privileged structures among NPs. Peculiar chemical features of the hit **2** were an alkoxy side chain and a hydroxyl group *ortho* to the carbonyl function (A-ring), while the B-ring was decorated with a 3,4,5-trimethoxy substitution pattern.

A predominant approach for the design of multitarget agents is the “pharmacophores combination” strategy, that involves the arrangement of different pharmacophore units from selected bioactive ligands to obtain analogues with an improved and expanded biological profile [19]. Accordingly, in this work, the main scaffold of the benzophenone **1** was combined with that of the chalcone **2**, which could also be considered a metal chelating pharmacophore that could allow to widen the spectrum of biological activities of the obtained conjugate (analogue **4**). To preliminarily explore the SAR of this new hybrid, some structural modifications were performed, involving the replacement of the electron rich 3,4,5-trimethoxy-phenyl ring with a rigid and planar naphthalene moiety (**5**), since these groups proved to be valuable functions in the study of the chemical space of some AD targets such as the acetylcholinesterase enzyme [20]. Moreover, to assess the importance of the chalcone *ortho*-hydroxy-*trans*-α,β-unsaturated carbonyl framework for BACE-1 inhibition, this function was cyclized to a flavanone nucleus (to obtain compounds **6** and **7**). Finally, applying a molecular simplification approach, the acetophenone-based derivative **3** was also included in the series. The new flavonoid-based conjugates were first tested on BACE-1 enzyme to assess their inhibitory potency. Then, the metal complexation potential of some selected candidates was also studied both *in vitro* and in solution. Finally, the ability to offer protection against metal induced oxidative stress was also investigated.

## 2. Results

### 2.1. Chemistry

**Scheme 1:**
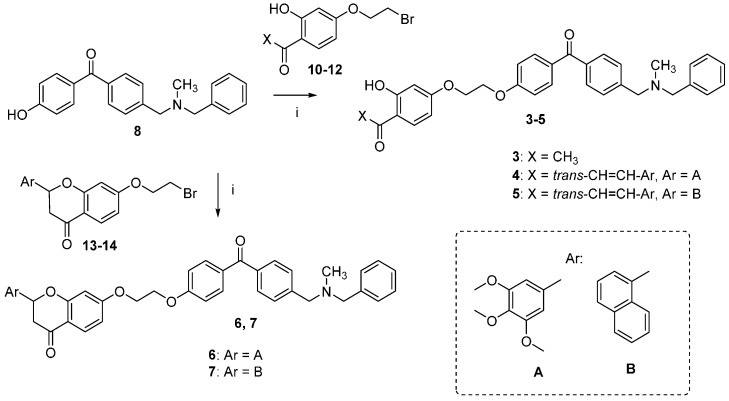
Synthesis of compounds **3**–**7**. *Reagents and Conditions*: (i) K_2_CO_3_, acetone, reflux.

**Scheme 2:**
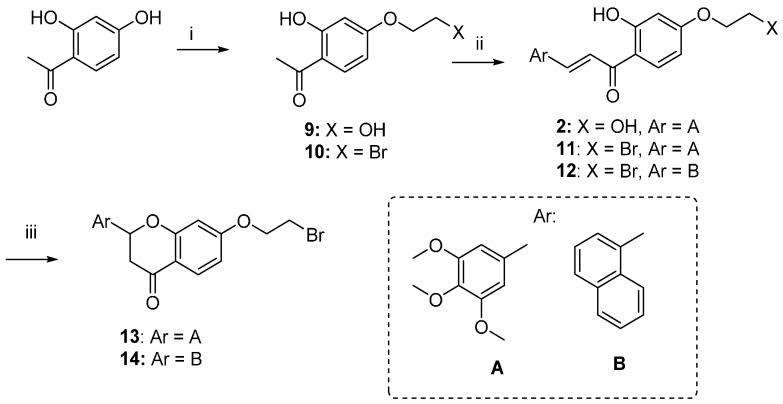
Synthesis of intermediates **10**–**14**. *Reagents and Conditions:* (i) 1-Bromoethanol or 1,2-dibromoethane, K_2_CO_3_, acetone, reflux; (ii) substituted benzaldehydes, KOH, EtOH, rt.; (iii) NaOAc, EtOH, 80 °C, reflux.

The synthetic route followed for the preparation of the chalcone-benzophenone conjugates **3**–**7** is depicted in Scheme 1. The hydroxylated benzophenone **8** was synthesized according to our previously described procedure [17] and then subjected to Williamson reactions with the different flavonoid-based derivatives **10**–**14**, using a parallel synthesis apparatus, to give the final compounds **3**–**7**. Intermediates **10**–**14** were obtained according to Scheme 2, in which 2,4-dihydroxyacetophenone was alkylated with 1,2-dibromoethane in hot acetone and in the presence of K_2_CO_3_ to obtain key intermediate **10**, that underwent a Claisen-Schmidt condensation reaction with the selected aldehydes and KOH, to produce the chalcone-based intermediates **11**,**12** in good yields (70%–90%); their cyclization to the corresponding (±) flavanones **13**,**14** was accomplished by treatment with sodium acetate in refluxing ethanol. When 2,4-dihydroxyacetophenone was alkylated with 1-bromo-ethanol under the abovementioned conditions, compound **9** was obtained, that underwent a Claisen-Schmidt condensation with 3,4,5-trimethoxybenzaldehyde to give the final compound **2**. Finally, compound **3** was obtained by Williamson reaction between **10** and **8**.

### 2.2. Biological Evaluation

The new benzophenone-based hybrids **3**–**7** presented in this study were first evaluated for their *in vitro* ability to inhibit the enzymatic activity of BACE-1, by means of a biochemical assay performed using the fluorescence resonance energy transfer (FRET) methodology [21] and in comparison to inhibitor IV [22] as reference compound (Table 1). For the most interesting compounds **3**–**5**, endowed with the *orto*-hydroxycarbonyl substitution pattern, the neuroprotective potential was determined as lack of toxic effects on neuronal cell viability and as Cu(II) chelating activity (Table 2); moreover, their direct interactions with Cu(II) ion were confirmed by a UV-vis spectrophotometric study. 

Finally, for the most interesting compound **5**, the ability to protect from oxidative stress following complexation with Cu(II) was studied.

#### 2.2.1. BACE-1 Inhibition

All the newly synthesized conjugates **3**–**7** showed good *in vitro* BACE-1 inhibitory potencies, higher than the starting hits; data are reported in Table 1. More in detail, when the benzophenone scaffold was linked to the flavonoid precursor acetophenone to give compound **3**, an encouraging result was obtained (IC_50_ = 3.18 µM). Indeed, the chalcone-benzophenone conjugates **4** and **5** also showed good BACE-1 inhibition, as their potencies were maintained in the same low-micromolar range as **3**. In particular **4**, bearing a 3,4,5-trimethoxyphenyl moiety, showed higher activity than the starting chalcone-based derivative **2**, and proved to be the most active of the series (IC_50_ = 1.06 µM), slightly more potent than **5** carrying a 1-naphthyl function (IC_50_ = 4.02 µM). The corresponding flavanone-benzophenone analogues **6** and **7**, respectively, maintained the same trend of inhibition, and again the 3,4,5-trimethoxyphenyl ring allowed compounds to achieve a more favorable enzyme inhibition with respect to the naphthyl ring.

#### 2.2.2. Neuroprotection

The neuroprotective potential was investigated for selected compounds **3**–**5** bearing the metal chelating pharmacophore (*ortho*-hydroxycarbonylaryl), by evaluating: (a) the neurotoxic effects; (b) the Cu (II) chelation ability; (c) the antioxidant properties.

##### Neurotoxicity

Cytotoxic effects in several *in vitro* or *in vivo* models have been reported for some chalcones [23]. Therefore, to discriminate the neurotoxic concentrations of the conjugates **3**–**5** we evaluated the cell viability of human neuronal SH-SY5Y cells after 24 h treatment with these conjugate at different concentrations (1.25–40 μM) using the MTT assay [24]. In our experimental conditions, neurotoxic effects were recorded for acetophenone **3** (IC_50_ = 10.89 ± 1.63 µM) and chalcone **4** (IC_50_ = 23.66 ± 3.54 μM) but not for chalcone **5**. Although, conjugate **4** showed some neurotoxic effects, its low-micromolar BACE-1 IC_50_ value suggests a reasonably favorable selectivity index SI (around 22). Thus, the presence of an α,β-unsatured carbonyl fragment seems to positively influence the degree of neuronal toxicity, leading to molecules endowed with a favorable SI.

##### In Vitro* Cu(II) Chelation*

Bathocuproinedisulfonic acid disodium salt (BCS) [25] assay was employed to assess the Cu(II) chelating ability of various concentration of conjugates **3**–**5** and the disodium salt of ethylene diaminetetraacetic acid, (Na_2_EDTA, 5–20 µM), in the presence of a fixed 40 µM dose of CuSO_4_. Data were expressed as chelating activity percentage (Table 2). The simplified analogue **3** showed only a modest affinity for Cu(II) (5 µM, 0.00%; 10 µM, 3.49% ± 1.71%; 20 µM, 5.37% ± 0.16%), that was improved by the presence in the molecule of the 3-aryl-propen-1-one framework, as in **4** (5 µM, 7.16% ± 0.24%; 10 µM, 10.77% ± 0.82%; 20 µM, 15.07% ± 1.32%) and **5** (5 µM, 9.48% ± 0.64%; 10 µM, 13.78% ± 1.74%; 20 µM, 19.73% ± 0.30%).

##### Total Antioxidant Activity

The total antioxidant capacity (TAC) of the selected conjugates **3**–**5**, at various concentrations (0.625–10 µM), was determined using the 2,2-diphenyl-1-picrylhydrazyl (DPPH) inhibition assay [24,26]. In this context, no direct antioxidant effect was recorded (data not shown). Taking into account all these preliminary results, conjugate **5,** endowed with the best neuronal compatibility and Cu(II) chelating ability, represents the most promising compound of the series, worth of further investigation regarding its neuroprotective action, in particular the mechanism of action by which **5** may exert its antioxidant activity.

##### Inhibition of Cu(II)-Mediated ROS Formation

**Figure 2:**
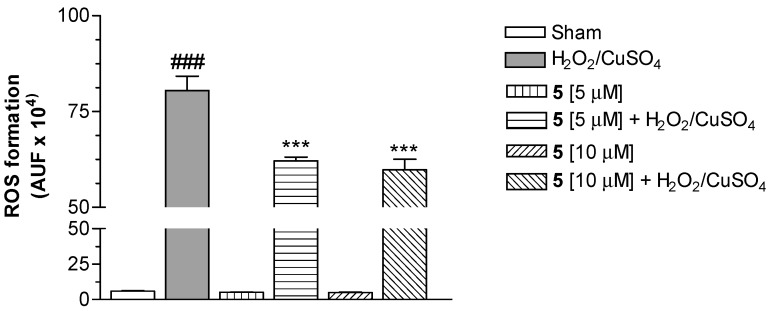
Effect of compound **5** on ROS formation elicited by H_2_O_2_/CuSO_4_ in SH-SY5Y cells. The cells were treated with various concentrations of **5** and H_2_O_2_ (100 µM)/CuSO_4_ (25 µM) for 30 min. At the end of the incubation, intracellular ROS formation was determined using a fluorescence probe, as described in the Materials and Methods section. The arbitrary units of fluorescence (AUF) values are shown as mean ± SD of at least three independent experiments (^###^ *P* < 0.001 versus untreated cells, *** *P* < 0.001 versus cells treated with H_2_O_2_/CuSO_4_, at ANOVA with Bonferroni’s *Post Hoc* Test).

Our attention was then focused on the study of the intracellular antioxidant effect mediated by Cu(II) chelation. Therefore, compound **5** was selected to be studied in SH-SH5Y cells for its ability to counteract ROS formation elicited by Fenton reaction with CuSO_4_ (25 µM) and H_2_O_2_ (100 µM), and using 2′,7′-dichlorodihydrofluorescein diacetate (H_2_DCF-DA) as the fluorescent probe [27]. As shown in Figure 2, the conjugate **5** at 5 and 10 µM significantly decreased the ROS formation evoked by H_2_O_2_/CuSO_4_, expressed as arbitrary fluorescence units (AUF). This finding suggests the ability of **5** to stop the Fenton reaction through the Cu(II) chelation.

### 2.3. Spectrophotometric Evaluation of Cu(II) Ion Complexation

**Figure 3:**
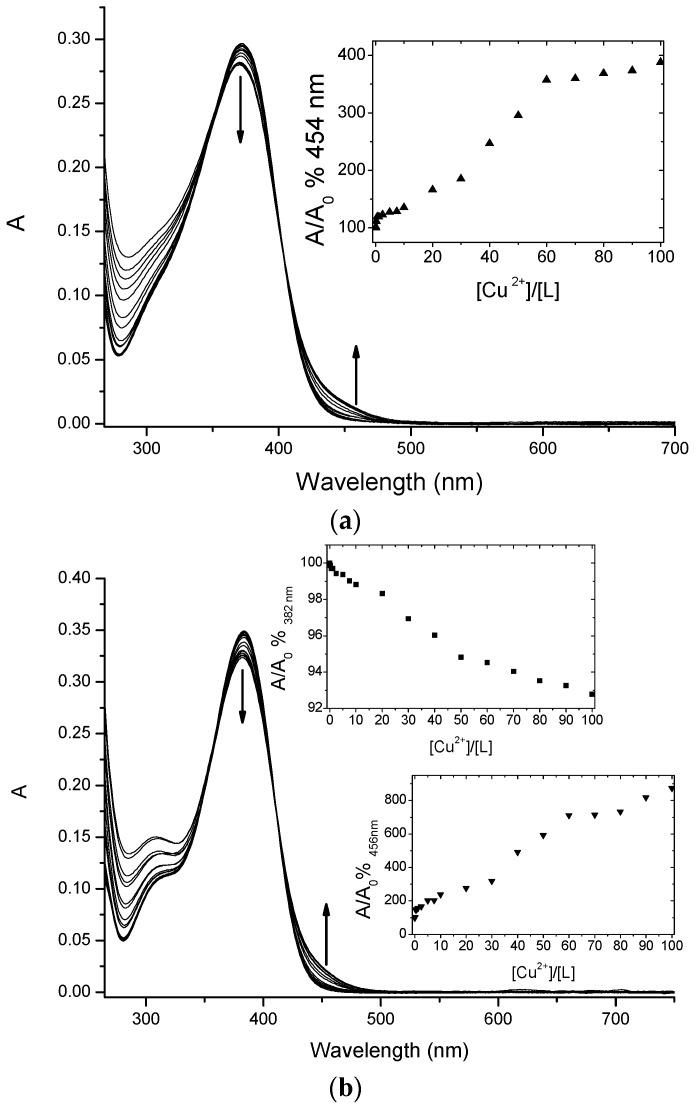
Titration curves of **4** (**a**) and **5** (**b**) (at 5 µM concentration) with increasing amounts of Cu(II) in MOPS 10 mM pH 7.4/DMF (1:4). Inset: absorbance ratio at 382 nm versus Cu(II) equivalents.

Chalcone conjugates **4** and **5** were investigated to evaluate their ability to complex Cu(II) ions via a UV-vis spectrophotometric study. The spectra, reported in Figure 3, showed that both compounds were able to chelate the selected ion.

Flavonoids, in general, and *ortho*-hydroxychalcones in particular, thanks to their weak acidic character, could be able to complex metal cations with more than one coordination site [11]. Therefore, their affinity for the different metal ions strongly depends on the medium and pH. To experimentally confirm via spectroscopic measurements the Cu(II) chelation effect observed in the physiological environment for the selected chalcone conjugates **4** and **5**, we had to choose a medium that could somehow mimic this very complex situation, taking into account the solubility constrains as well. In alkaline media there is maximum deprotonation, but precipitation equilibria of Cu(II) hydroxide cannot be easily excluded and controlled.

Therefore, we decided to carry on the spectrophotometric measurements in a mixed solvent: aqueous MOPS aqueous buffer 1 × 10^−2^ M pH 7.4 /DMF in 1:4 ratio, respectively. Flavonoids bearing the 4-carbonyl-5-hydroxyl chelation site have characteristic UV-vis spectra, characterized by a wide and non-structured band centred around 370–380 nm, with a molar extinction coefficient of 50,000 cm^−1^·M^−1^ that is attributed to HOMO-LUMO transition with a π-π* character.

The addition of increasing amounts of Cu(II) ions in solution caused a decrease of the maximum of this band for both ligands, with a concomitant increase of the absorbance around 450 nm. These changes are quite small, but in line with other similar systems already reported in the literature, and therefore diagnostic for the copper complex formation [28].

**Figure 4:**
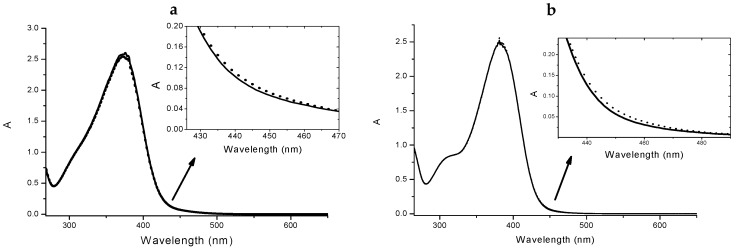
Absorption spectra of **4** 4 × 10^−5^ M in absence (solid line) and in presence (dotted line) of 2.5 × 10^−5^ M Cu(II) (**a**). Inset: spectral detail of the changing upon the addiction of the metal. Absorption spectra of **5** 4 × 10^−5^ M in absence (solid line) and in presence (dotted line) of 2.5 × 10^−5^ M Cu(II) (**b**). Inset: spectral detail.

Chalcones **4** and **5** underwent a UV-vis spectrophotometric study to evaluate their ability to complex Cu(II) ions and the spectra were reported in Figure 3 and Figure 4. It is important to underline that chalcones, being multi-site metal cation binders, can generate complexes with different stoichiometries and/or structures, thus it is difficult to discriminate among them only via spectrophotometric measurements. Nevertheless, for both selected compounds during the titration procedure we observed the formation of an isosbestic point (402 nm for **4** and 410 nm for **5**, Figure 3a,b, respectively); this feature is a clear indication that the complexation is taking place, even if it does not exclude multiple equilibria involving different metal to ligand stoichiometries (as possibly suggested by the profile of the inset of Figure 3).

Furthermore, from the titration data an association constant was estimated that was of the order of magnitude of 10^4^ for a 1:1 complex stoichiometry. Undoubtedly, the complex cellular environment could drastically influence the binding events, thus any structure prediction could be very difficult; however, the study of the interactions performed in a simpler but similar surrounding could offer very informative experimental evidences. In the light of these considerations, we have studied three different concentrations of each ligand, reproducing the conditions of the biological study. As expected from the titration results, for both **4** and **5** we observed the formation of the complexes as shown in Figure 4 (data not shown for the other concentrations).

## 3. Discussion

Molecules able to modulate a single protein target prove to be ineffective for treatement of multifactorial and complex disorders such as AD. Researchers are now turning to the design of multitarget compounds, single chemical entities able to simultaneously modulate several molecular targets or pathways involved in the disease, since these multipotent compounds could enable to achieve a better efficacy profile compared to the single-target agents. Indeed BACE-1, catalyzing the rate-limiting step in the generation of Aβ fragments, decreases the formation of Aβ. On the other hand, Aβ is a metalloprotein that contains metal binding sites, and metals such as copper are present at elevated concentrations in Aβ plaques and can promote the oligomerization of Aβ, increasing its cytotoxicity. In this scenario, the “pharmacophores combination” design strategy was applied in this study and two naturally inspired privileged structures, identified as weak BACE-1 inhibitors, were connected. In particular, a benzophenone-based scaffold was conjugated to flavonoid-related frameworks, namely chalcone and flavanone, as metal chelating pharmacophores. The small library of hybrid molecules **3**–**7** turned out to inhibit BACE-1 enzyme with a low-micromolar potency and the new compounds proved to be more active than the starting hit fragments. Moreover, the chalcone-based analogues **4** and **5**, endowed with an α,β-unsaturated carbonyl fragment, showed an interesting biological profile in terms of ability to chelate Cu(II) ions, associated with a lack of neurotoxic effects (favorable selectivity index).

Indeed redox active Cu(II) in complex with Aβ can generate H_2_O_2_, which may be converted to hydroxyl radicals via Fenton reaction, resulting in oxidative cellular damage. Taking this into account, **5** was also tested as an antioxidant and proved to be able to counteract the Cu(II)-dependent ROS formation.

Finally, compound **5** proved to be a valuable multifunctional compound, able to protect from common AD pathogenic events such as Aβ formation and Cu injury. It is thus characterized by a low-micromolar inhibitory potency on BACE-1 enzyme, a good Cu (II) chelating ability (confirmed also by UV-vis spectrophotometric studies) and a consequent efficacy to protect from Cu(II)-mediated toxicity. In summary, our initial design hypothesis was validated by the multipotent biological profile of the benzophenone-chalcone conjugate **5** that represents a new promising lead compound, worth of being further optimized and investigated in terms of structure-activity relationships (SAR). The results of this study can be considered as the basis for the design of new small libraries of hybrids obtained by conjugation of biologically relevant frameworks: the *N*,*N*’-benzylmethylamino-benzophenone scaffold on one side and flavonoid-related frameworks on the other side.

## 4. Materials and Methods

### 4.1. General Information

Starting materials, unless otherwise specified, were high-purity commercial products. Solvents were of analytical grade. Melting points were determined in open glass capillaries, using a Büchi apparatus and are uncorrected. ^1^H-NMR and ^13^C-NMR spectra were recorded on a Gemini spectrometer (400 MHz Varian, Palo Alto, CA, USA) and chemical shift are reported as parts per million (ppm value) relative to the peak for tetramethylsilane (TMS) as internal standard. Standard abbreviations indicating spin multiplicities are given as follows: s (singlet), d (doublet), t (triplet), br (broad), q (quartet) or m (multiplet). Mass spectra were recorded on a ZQ 4000 apparatus (Waters, Milford, MA, USA) operating in electrospray mode (ES). Chromatographic separations were performed on silica gel columns by the flash method (Kieselgel 40, 0.040–0.063 mm, Merck, Darmstadt, Germany). Reactions were followed by thin layer chromatography (TLC) on precoated silica gel plates (Merck Silica Gel 60 F254) and then visualized with a UV lamp. The purity of the tested compounds was determined by HPLC analysis, performed on a LC 1500 PU-1587 system (Jasco, Easton, MD, USA); the column used was a Luna C18(2) 5 m 4.60 mm × 150 mm (Phenomenex, Castel Maggiore (BO), Italy); elution conditions: H_3_PO_4_ 0.5%/ACN (75:25, *v*/*v*) the flow-rate was 1.2 mL/min and the injection volume was 5 μL; peaks were detected at 254 nm and results were >98% purity. Compounds were named relying on the naming algorithm provided in ChemDraw Professional 15.0, developed by CambridgeSoft Corporation (Waltham, MA, USA).

### 4.2. Chemistry

#### 4.2.1. General Procedure for the Synthesis of **3**–**7**, **9**, **10** by Williamson Reaction

To a room temperature solution of the phenol derivative (1.0 mmol) in acetone (10 mL) K_2_CO_3_ (1.1 mmol) was added, followed by the corresponding bromoalkyl derivative (1.0 mmol). The reaction mixture was heated under reflux and the reaction progress was monitored by TLC. Upon reaction completion, the mixture was hot filtered, and the solvent was evaporated under reduced pressure. The resulting crude product was purified by column chromatography over a silica gel column using a mixture of petroleum ether/ethyl acetate as the solvent system to give the desired pure products.

*1-[2-Hydroxy-4-(2-hydroxyethoxy)phenyl]ethanone* (**9**). Reaction of 2,4-dihydroxyacetophenone (5.0 mmol, 0.76 g) and 2-bromoethanol (5.5 mmol, 0.69 g, 0.39 mL) gave the crude product that was purified by flash chromatography (petroleum ether/ethyl acetate 7/3).Yield = 85%, mp: 64–66 °C. ^1^H-NMR (CDCl_3_) δ 2.56 (s, 3H, COCH_3_), 3.95–4.01 (m, 2H, CH_2_OH), 4.10–4.14 (m, 2H, OCH_2_), 6.40 (d, *J* = 1.8 Hz, 1H, ArH-3), 6.50 (dd, *J* = 1.8 and 8.6 Hz, 1H, ArH-5), 7.64 (d, *J* = 8.6 Hz, 1H, ArH-6).

*1-[2-Hydroxy-4-(2-bromoethoxy)phenyl]ethan-1-one* (**10**). Reaction of 2,4-dihydroxyacetophenone (5.0 mmol, 0.76 g) and 1,2-dibromoethane (5.0 mmol, 0.94 g, 0.45 mL), gave the crude product that was purified by flash chromatography (petroleum ether/ethyl acetate 4:1).Yield = 85%, mp: 61–63 °C. ^1^H-NMR δ: 2.60 (s, 3H, COCH_3_), 3.83 (t, 2H, *J* = 6.00 Hz, CH_2_Br), 4.26 (t, 2H, *J* = 6.00 Hz, OCH_2_), 6.40 (d, *J* = 2.2 Hz, 1H, ArH-3) 6.48 (dd, *J* = 8.0 and 2.2 Hz, 1H, ArH-5), 7.65 (d, 1H, *J* = 9.20 Hz, ArH-6).

*1-{4-[2-(4-4-Benzylmethylaminomethylbenzoylphenoxy)ethoxy]-2-hydroxyphenyl}ethan-1-one* (**3**)*.* Reaction of benzophenone (Bp) **8** (1.0 mmol, 0.33 g) and **10** (1.1 mmol, 0.28 g) gave a crude product that was purified by flash chromatography (CH_2_Cl_2_/CH_3_OH 9.5:0.5) to give a semisolid compound, Yield = 51%, HCl; mp: 264–268 °C. ^1^H-NMR (400 MHz, CDCl_3_) δ 2.33 (s, 3H, NCH_3_), 2.55 (s, 3H, COCH_3_), 3.56 (s, 2H, NCH_2_), 3.59 (s, 2H, NCH_2_), 3.90–3.94 (m, 4H, OCH_2_CH_2_O), 6.48 (d, *J* = 2.2 Hz, 1H, Ch-Ar), 6.51 (dd, *J* = 8.0 and 2.2 Hz, 1H, Ch-Ar), 7.00 (d, 2H, *J* = 8.40 Hz, Bf-Ar), 7.26–7.46 (m, 5H, Bn-Ar), 7.49 (d, 2H, *J* = 8.60 Hz, Bf-Ar), 7.63 (d, 1H, *J* = 8.0 Hz, Ch-Ar), 7.75 (d, 2H, *J* = 8.60 Hz, Bf-Ar), 7.85 (d, 2H, *J* = 8.60 Hz, Bf-Ar). ^13^C-NMR (101 MHz, CDCl_3_): δ 26.64, 61.055, 61.62, 64.34, 64.66, 103.87, 106.98, 113.44, 126.90, 128.12, 128.52, 128.74, 128.86, 129.77, 130.41, 132.41, 136.91, 138.22, 143.69, 162.59, 165.11, 166.65, 197.56, 202.61. (ESI^+^) 510 *m*/*z*: [M + H]^+^.

*E-1-{4-[2-(4-4-Benzylmethylaminomethylbenzoylphenoxy)ethoxy]-2-hydroxyphenyl}-3-(3,4,5-trimethoxy-phenyl)prop-2-en-1-one* (**4**). Reaction of Bp **8** (1.0 mmol, 0.33 g) and chalcone (Ch) **11** (1.1 mmol, 0.48 g) gave a crude product that was purified by flash chromatography (CH_2_Cl_2_/CH_3_OH 9.25:0.75) to give a semisolid compound. Yield = 65%, HCl; mp: 220–222 °C. ^1^H-NMR (400 MHz, CDCl_3_) δ 2.30 (s, 3H, NCH_3_), 3.57 (s, 2H, NCH_2_), 3.59 (s, 2H, NCH_2_), 3.91 (s, 3H, OCH_3_), 3.94 (s, 6H, OCH_3_), 4.20–4.34 (m, 4H, OCH_2_CH_2_O), 6.50 (d, *J* = 2.2 Hz, 1H, Ch-Ar), 6.54 (dd, *J* = 8.0 and 2.2 Hz, 1H, Ch-Ar), 6.87 (s, 2H, Ch-Ar), 7.00 (d, 2H, *J* = 8.60 Hz, Bp-Ar), 7.16–7.36 (m, 5H, Bn-Ar), 7.26 (d, 1H, *J* = 15.60 Hz, Ch-βCH=CH), 7.72 (d, 2H, *J* = 8.60 Hz, Ch-Ar), 7.78 (d, 1H, *J* = 8.60 Hz, Bp-Ar), 7.85 (d, 1H, *J* = 15.60 Hz, Ch-αCH=CH), 7.88 (d, 2H, *J* = 8.60 Hz, Bp-Ar); ^13^C-NMR (101 MHz, CDCl_3_): δ 56.11, 60.85, 64.34, 64.66, 103.86, 106.93, 113.45, 118.74, 126.96, 128.17, 128.56, 128.71, 128.80, 129.70, 130.46, 132.45, 136.92, 138.24, 143.68, 145.32, 153.01, 153.22, 162.5, 165.11, 166.62, 197.58, 192.41. (ESI^+^) *m*/*z*: 687 [M + H]^+^.

*E-1-{4-[2-(4-4-Benzylmethylaminomethylbenzoylphenoxy)ethoxy]-2-hydroxyphenyl}-3-(naphthalen-1-yl)-prop-2-en-1-one* (**5**)*.* Reaction of Bp **8** (1.0 mmol, 0.33 g) and Ch **12** (1.1 mmol, 0.44 g) gave a crude product that was purified by flash chromatography (CH_2_Cl_2_/CH_3_OH 9.25:0.75) to give a semisolid compound. Yield = 51%, HCl: mp: 197–99 °C. ^1^H-NMR (400 MHz, CDCl_3_) δ 2.32 (s, 3H, NCH_3_), 3.59 (s, 2H, NCH_2_), 3.62 (s, 2H, NCH_2_), 4.30–4.40 (m, 4H, OCH_2_CH_2_O), 6.55 (d, *J* = 2.2 Hz, 1H, Ch-Ar), 6.58 (dd, *J* = 8.0 and 2.2 Hz, 1H, Ch-Ar), 7.02 (d, 2H, *J* = 8.60 Hz, Bf-Ar), 7.20–7.61 (m, 10H, Ar), 7.75 (d, 1H, *J* = 16.4 Hz, β-CH=CH), 7.79–7.97(m, 8H, Ar), 8.03 (d, 1H, *J* = 8.0 Hz, Ch-Ar), 8.75 (d, 1H, *J* = 15.60 Hz, αCH=CH); ^13^C NMR (101 MHz, CDCl_3_): δ 61.05, 61.62, 64.34, 64.66, 103.88, 106.96, 113.44, 121.33, 122.85, 126.14, 126.99, 128.19, 128.51, 128.76, 128.83, 129.72, 130.43, 132.48, 132.47, 133.54, 135.54, 136.94, 138.23, 143.63, 162.59, 165.11, 166.65, 197.50, 192.81. (ESI^+^) *m*/*z*: 648 [M + H]^+^.

*7-{2-[4-(4-Benzylmethylaminomethylbenzoyl)phenoxy]ethoxy}-2-(3,4,5-trimethoxyphenyl)chroman-4-one* (**6**). Reaction of Bp **8** (1.0 mmol, 0.33 g) and flavone (Fl) **13** (1.1 mmol, 0.48 g) gave a crude product that was purified by flash chromatography (CH_2_Cl_2_/CH_3_OH 9.5:0.5) to give a semisolid compound. Yield = 59%, HCl: mp: 182–94 °C. ^1^H-NMR (400 MHz, CDCl_3_) δ 2.32 (s, 3H, NCH_3_), 2.87 (dd, 1H, *J* = 16.5 and 2.8 Hz, H3a-Fl), 3.06 (dd, 1H, *J* = 16.5 and 13.1 Hz, H3b-Fl) 3.70 (s, 2H, NCH_2_), 3.74 (s, 2H, NCH_2_), 3.87 (s, 3H, OCH_3_),3.90 (s, 6H, OCH_3_), 4.40–4.46 (m, 4H, OCH_2_CH_2_O), 5.37 (dd, 1H, *J* = 13.1 and 2.8 Hz, H2-Fl), 6.57 (d, *J* = 2.2 Hz, 1H, Fl-Ar), 6.70 (dd, *J* = 8.0 and 2.2 Hz, 1H, Fl-Ar), 6.75 (s, 2H, Fl-Ar), 7.00 (d, 2H, *J* = 8.60 Hz, Bf-Ar), 7.26–7.36 (m, 5H, Bn-Ar), 7.78 (d, 1H, *J* = 8.60 Hz, Bf-Ar), 7.81 (d, 2H, *J* = 8.60 Hz,Bf-Ar), 7.86 (d, 2H, *J* = 8.60 Hz,Bf-Ar); ^13^C-NMR (101 MHz, CDCl_3_): δ 42.45, 56.10, 59.21, 61.05, 61.62, 64.34, 64.66, 83.44, 101.74, 103.86, 106.93, 113.46, 126.98, 128.12, 128.54, 128.76, 128.82, 129.78, 130.45, 132.43, 134.91, 136.92, 138.22, 143.67, 153.21, 138.74, 163.25, 165.11, 166.65, 197.51, 190.21. MS (ESI^+^) *m*/*z*: 689 [M + H]^+^.

*7-{2-[4-(4-(Benzylmethylaminomethylbenzoyl)phenoxy]ethoxy}-2-(naphthalen-1-yl)chroman-4-one* (**7**). Reaction of Bf **8** (1.0 mmol, 0.33 g) and Fl **13** (1.1 mmol, 0.44 g) gave a crude product that was purified by flash chromatography (CH_2_Cl_2_/CH_3_OH 9.5:0.5) to give a semisolid compound. Yield = 45%, HCl: mp: 172–95 °C. ^1^H-NMR (400 MHz, CDCl_3_) δ 2.22 (s, 3H, NCH_3_), 3.03 (dd, 1H, *J* = 16.5 and 2.8 Hz, H3a-Fl), 3.26 (dd, 1H, *J* = 16.5 and 13.1 Hz, H3b-Fl) 3.54 (s, 2H, NCH_2_), 3.61 (s, 2H, NCH_2_), 4.36–4.48 (m, 4H, OCH_2_CH_2_O), 6.21 (dd, 1H, *J* = 13.1 and 2.8 Hz, H2-Fl), ), 6.57 (d, *J* = 2.2 Hz, 1H, Fl-Ar), 6.58 (dd, *J* = 8.0 and 2.2 Hz, 1H, Fl-Ar), 7.02 (d, 2H, *J* = 8.60 Hz, Bf-Ar), 7.20–7.61 (m, 10H, Ar), 7.79–7.97(m, 8H, Ar), 8.05 (d, 1H, *J* = 8.0 Hz, Fl-Ar). ^13^C-NMR (101 MHz, CDCl_3_): δ 43.21, 61.05, 61.62, 64.34, 64.66, 80.80, 103.81, 106.93, 113.09, 113.43, 125.47, 126.41, 126.97, 128.17, 128.53, 128.78, 128.89, 129.79, 130.49, 132.45, 134.98, 136.31,136.95, 138.21, 136.88, 143.63, 162.57, 163.25, 165.11, 166.65, 197.57, 190.66. MS (ESI^+^) *m*/*z*: 649 [M + H]^+^.

#### 4.2.2. General Procedure for the Synthesis of Chalcones **2**, **11**, **12** by Claisen-Schmidt Reaction

To a solution of acetophenone (1.0 mmol) and substituted benzaldehyde (1.1 mmol) in ethanol (10 mL), 50% KOH aqueous solution (3.0 mmol, 0.11 mL) was added dropwise. The reaction mixture was stirred at r.t. overnight, then diluted with water and acidified with 6 N HCl. The solid formed was collected by *vacuum* filtration and the crude residue was purified by flash column chromatography or by crystallization from ethanol.

*(E)-1-[2-Hydroxy-4-(2-hydroxyethoxyphenyl)]-3-(3,4,5-trimethoxyphenyl)prop-2-en-1-one* (**2**). Reaction of **9** (1.0 mmol, 0.19 g) and 3,4,5-trimethoxybenzaldehyde (1.1 mmol, 0.22 g) gave a crude that was purified by flash chromatography (petroleum ether/ethyl acetate 7:3) to afford **2** as a yellowish solid. Yield = 71%, mp: 186–188 °C. ^1^H-NMR (CDCl_3_) δ 3.91 (s, 3H, OCH_3_), 3.94 (s, 6H, OCH_3_), 4.00 (t, *J* = 6.0 Hz, 2H, CH_2_OH), 4.13 (t, *J* = 6.0 Hz, 2H, OCH_2_), 6.47 (d, *J* = 2.2 Hz, 1H and H3), 6.52 (dd, *J* = 2.2 and 8.2 Hz, 1H and H5), 6.87 (s, 2H, H2’ and H6’), 7.45 (d, *J* = 15.4 Hz, 1H, βCH=CH), 7.78 (d, *J* = 15.4 Hz, 1H, αCH=CH), 7.84 (d, 1H, *J* = 8.6 Hz, H6). ^13^C-NMR (CDCl_3_) δ 56.23, 56.28, 60.33, 66.43, 79.51, 101.74, 105.81, 118.35, 118.44, 121.09, 121.26, 136.63, 136.66, 145.41, 147.48, 165.90, 166.64, 192.85. MS (ESI^+^) *m*/*z*: 375 [M + H] ^+^.

*(E)-1-[4-(2-Bromoethoxy)-2-hydroxyphenyl]-3-(3,4,5-trimethoxyphenyl)prop-2-en-1-one* (**11**). Reaction of **10** (1.0 mmol, 0.26 g) and 3,4,5-trimethoxybenzaldehyde (1.1 mmol, 0.22 g) gave a crude that was purified by crystallization from ethanol to afford **11** as a yellowish solid. Yield = 91%, mp: 170–172 °C. ^1^H-NMR δ 3.35 (t, 2H, *J* = 6.6, CH_2_-Br), 3.80 (s, 3H, OCH_3_), 3.84 (s, 6H, OCH_3_), 4.15 (t, 2H, *J* = 6.6 Hz, OCH_2_), 6.45 (d, 1H, *J* = 2.2 Hz, H3), 6.54 (dd,1H, *J* = 2.2 and 8.2 Hz, H5), 6.88 (s, 2H, H2’ and H6’), 7.43 (d, 1H, *J* = 15.6 Hz, βCH=CH), 7.82 (d, *J* = 15.6 Hz, 1H, αCH=CH), 7.88 (d, 1H, *J* = 8.6 Hz, H6).

*(E)-1-(4-(2-Bromoethoxy)-2-hydroxyphenyl)-3-(naphthalen-1-yl)prop-2-en-1-one* (**12**). Reaction of **10** (1.0 mmol, 0.26 g) and 1-naphthaldehyde(1.1 mmol, 0.16 g) gave a crude that was purified by crystallization from ethanol to afford **12** as a yellowish solid. Yield 89%, mp: 140–142 °C. ^1^H-NMR δ 3.36 (t, 2H, *J* = 6.8, CH_2_-Br), 4.18 (t, 2H, *J* = 6.8 Hz, OCH_2_), 6.46 (d, 1H, *J* = 2.2 Hz, H3), 6.51 (dd, 1H, *J* = 2.2 and 8.2 Hz, H5), 7.51–7.64 (m, 3H, Ar), 7.65 (d, 1H, *J* = 15.3 Hz, βCH=CH), 7.86–7.96 (m, 4H, Ar), 7.87 (d, 1H, *J* = 8.6 Hz, H6), 8.25 (d, *J* = 15.6 Hz, 1H, αCH=CH).

#### 4.2.3. General Procedure for the Synthesis of Flavanones **13**, **14**.

Chalcone (0.5 mmol) and sodium acetate (5.0 mmol) were dissolved in ethanol (10 mL) and the solution was heated under reflux for 24 h. The reaction mixture was allowed to cool to r.t. and then poured into ice/water (100 mL), and extracted with CH_2_Cl_2_ (3 × 30 mL). The combined organic layers were washed with brine, dried over Na_2_SO_4_, and concentrated under reduced pressure. The crude residue was purified by column chromatography using petroleum ether/ethyl acetate (7:3) as solvent system, to afford the pure flavanone.

*7-(2-Bromoethoxy)-2-(3,4,5-trimethoxyphenyl)chroman-4-one* (**13**). From the reaction of **11** (0.5 mmol, 0.20 g) and NaOAc (5.0 mmol, 0.04 g), **13** was obtained as pale yellow solid. Yield 48%, mp: 132–133 °C. ^1^H-NMR δ 2.89 (dd, 1H, *J* = 16.5 and 2.8 Hz, H3a), 3.11 (dd, 1H, *J* = 16.5 and 13.1 Hz, H3b), 3.35 (t, 2H, *J* = 6.6, CH_2_-Br), 4.26 (t, 2H, *J* = 6.6 Hz, OCH_2_),5.51 (dd, 1H, *J* = 13.1 and 2.8 Hz, H2), 6.43 (d, 1H, *J* = 2.2 Hz, H3), 6.57 (dd,1H, *J* = 2.2 and 8.2 Hz, H5), 6.91 (s, 2H, H2’ and H6’), 7.8 (d, 1H, *J* = 8.6 Hz, H6).

*7-(2-Bromoethoxy)-2-(naphthalen-1-yl)chroman-4-one* (**14**). From the reaction of **12** (0.5 mmol, 0.20 g) and NaOAc (5.0 mmol, 0.04 g), **14** was obtained as pale yellow solid. Yield 64%, mp: 115–117 °C. ^1^H-NMR δ 2.84 (dd, 1H, *J* = 16.5 and 2.8 Hz, H3a), 3.05 (dd, 1H, *J* = 16.5 and 13.1 Hz, H3b), 3.37 (t, 2H, *J* = 6.6, CH_2_-Br), 4.21 (t, 2H, *J* = 6.6 Hz, OCH_2_), 5.48 (dd, 1H, *J* = 13.1 and 2.8 Hz, H2), 6.53 (dd,1H, *J* = 2.2 and 8.2 Hz, H5), 7.54–7.68 (m, 3H, Ar), 7.85–8.01 (m, 4H, Ar), 7.88 (d, 1H, *J* = 8.6 Hz, H6).

### 4.3. BACE-1 Inhibition: FRET Inhibition Assay

Inhibition studies were performed using the following procedure: 5 μL of test compound (or DMSO) were pre-incubated with 175 μL of enzyme (10.37 nM, final concentration) in 20 mM NaOAc pH 4.5 containing CHAPS (0.1% *w*/*v*) for 1 h at r.t.; M-2420 (3 μM, final concentration) was then added and left to react for 15 min. The fluorescence signal was read at λ_em_ = 405 nm (λ_exc_ = 320 nm). DMSO concentration in the final mixture was maintained below 5% (*v*/*v*) to guarantee no significant loss of enzyme activity.Fluorescence intensities with and without inhibitor were compared and the percent inhibition due to the presence of test compounds was calculated. The background signal was measured in control wells containing all the reagents, except theenzymeand subtracted. The % inhibition due to the presence of increasing test compound concentration was calculated by the following expression: 100 − (IF_i_/IF_o_ × 100) where IF_i_ and IF_o_ are the fluorescence intensities obtained for the enzyme in the presence and in the absence of inhibitor, respectively. Inhibition curves were obtained for each compound by plotting the % inhibition versus the logarithm of inhibitor concentration in the assay sample. The linear regression parameters were determined and the IC_50_ extrapolated (GraphPad Prism 4.0, GraphPad Software Inc., San Diego, CA, USA).

### 4.4. Cu(II) Chelating Assay

The Cu(II) chelating activity of the compounds was determined using the bathocuproinedisulfonic acid disodium salt (BCS) assay as previously described [25]. Briefly, 50 μL of a 0.25 mM CuSO_4_ solution were mixed for 2 min with 50 μL of 5–20 µM compound solution in 100 µL of HEPES buffer (15 mM). Hydroxylamine (1 mM) was added after 2 min in order to reduce non-chelated cupric ions into cuprous ones. Finally, 50 μL of BCS (5 mM) or water (blank) was added and the absorbance at 490 nm was measured by spectrophotometry using a multilabel plate reader (VICTOR™ *X*3, PerkinElmer, Waltham, MA, USA) immediately and at 5 min. Na_2_EDTA (5–20 µM) was used as a positive control. The ability of the test compounds to chelate cupric ions was calculated using the following equation: chelating activity (%) = [1 − (Abs _BCS + compound_/Abs _BCS_) × 100].

### 4.5. Cell Cultures

Human neuronal (SH-SY5Y) cells were routinely grown in Dulbecco’s modified Eagle’ Medium (DMEM) supplemented with 10% fetal bovine serum, 2 mmol/l-glutamine, 50 U·mL^−1^penicillin and 50 μg·mL^−1^streptomycin at 37 °C in a humidified incubator with 5% CO_2_. 

### 4.6. Determination of Neurotoxicity

To evaluate the neurotoxicity of compounds, the SH-SY5Y cells were seeded in 96-well plates at 2 × 10^4^ cells/well, incubated for 24 h and subsequently treated with various concentrations of compounds (1.25–40 µM) for 24 h at 37 °C in 5% CO_2_;. The cell viability in terms of mitochondrial metabolic function was evaluated by the reduction in 3-(4,5-dimethyl-2-thiazolyl)-2,5-diphenyl-2*H*-tetrazolium bromide (MTT) to formazan as previously described [27]. The quantity of formazan was directly proportional to the number of viable cells. Briefly, the treatment medium was replaced with MTT (5 mg/mL) in phosphate-buffered saline (PBS) for 2 h at 37 °C in 5% CO_2_. After washing with PBS, the formazan crystals were dissolved with isopropanol. The amount of formazan was measured (570 nm, reference filter 690 nm) using a multilabel plate reader (VICTOR™ X3, PerkinElmer, Waltham, MA, USA). The neurotoxicity is expressed as concentration of compound resulting in 50% inhibition of cell viability.

### 4.7. Determination of Antioxidant Activity

The total antioxidant activity of the compounds was determined using the stable DPPH radical [24]. Briefly, 150 µL of 100 µM DPPH in ethanol was added to 50 µL of compound at different concentrations (0.625–10 µM) in 96 well plate. The absorbance of the reaction mixture was measured at 490 nm on a multilabel plate reader (VICTOR™ X3) after 1 min, 15 min and 30 min. The values was expressed as optical density of DPPH solution in absence or presence of compound.

The intracellular antioxidant activity of the compounds was evaluated in SH-SY5Y cells as previously described with minor changes [27]. Briefly, the SH-SY5Y cells were seeded in 96-well plates at 3 × 10^4^ cells/well and incubated for 24 h at 37 °C in 5% CO_2_. Subsequently cell culture medium was removed and 100 µL of 2′-7′dichlorodihydrofluorescein diacetate, H_2_DCF-DA, (10 µg/mL) were added to each wells. After 30 min of incubation at room temperature the H_2_DCF-DA solution was replaced with a solution of compound [5 and 10 µM] and H_2_O_2_ (100 µM)/CuSO_4_ (25 µM). The ROS formation was measured (excitation at 485 nm and emission at 535 nm) using a multilabel plate reader (VICTOR™ X3). The values are expressed as arbitrary fluorescence units (AUF).

### 4.8. Spectrophotometricdetermination of Cu(II) Chelation

All reagents and solvents were used as received (from Aldrich, St. Louis, MO, USA) without further purification: copper(II) sulphate hydrate (≥98%), 3-(*N*-Morpholino)propanesulfonic acid (MOPS, ≥99.5%), sodium hydroxide (≥98%), *N*,*N*-dimethylformamide (spectroscopic grade, DMF). A Milli-Q system (Millipore, Billerica, MA, USA) was used for the purification of water (resistivity ≥18.2 MΩ). MOPS bufferstock solution were obtained by a dissolution in Milli-Q water of 3-(*N*-morpholino)propane sulphonic acid to a final concentration of 10^−3^ M. The pH was corrected with a solution of sodium hydroxide 1 N to a value of pH 7.4. Absorption spectra were recorded on a Perkin-Elmer Lambda 45 spectrophotometer at room temperature. Spectrophotometric titrations were performed as follows: stock solutions of ligands (*ca.* 10^−3^ M) were prepared in DMF, diluted down to the desired concentration, in the quartz cuvette, in MOPS buffer 10^−3^ M pH 7.4/DMF (1:4) to a final volume of 2.5 mL and then titrated by adding increasing amounts of Cu(II) (3.04 × 10^−3^ Min DMF).

## Figures and Tables

**Table 1:** BACE-1 inhibitory potency of the new compounds **3**–**7** and reference compound (inhibitor IV).

Compound	R	*h*BACE-1 Inhibition IC_50_ (μM) ^a^
**3**	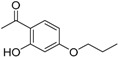	3.18 ± 0.21
**4**	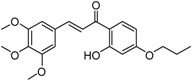	1.06 ± 0.15
**5**	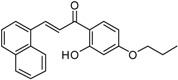	4.02 ± 0.29
**6**	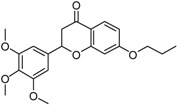	1.56 ± 0.23
**7**	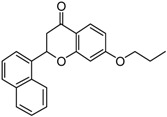	3.97 ± 0.33
Inhibitor IV ^b^		0.02 ± 0.00

^a^ All data were obtained at least in triplicate, and the values listed are the average from multiple experiments, each performed in triplicate. SEM ± standard error of the mean; ^b^ See ref [22].

**Table 2:** Neurotoxicity and Cu(II) chelating activity of the studied compounds and positive control Na_2_EDTA.

Compound	Cell Viability IC_50_ (µM) ^a^	Cu(II)-Chelating Activity (%) ^b^
**3**	10.89 ± 1.63	5.37 ± 0.16
**4**	23.66 ± 3.54	15.07 ± 1.32
**5**	n.d ^c^	19.73 ± 0.30
Na_2_EDTA	-	17.10 ± 1.44

^a^ Concentration of compound resulting in 50% inhibition of cell viability after 24 h treatment. The values are mean ± SD of at least two independent experiments; ^b^ Chelating activity observed at 20 µM concentration of compound and Na_2_EDTA as positive control. The values are mean ± SD of at least two independent experiments; ^c^ n.d. = IC_50_ not determined because less than 50% inhibition was observed at the highest concentration tested (40 µM).

## Abbreviations

The following abbreviations are used in this manuscript:

AD: Alzheimer’s disease
Aβ: amyloid β
APP: amyloid β protein precursor
BACE-1: β-site APP cleaving enzyme-1
BBB: blood brain barrier
Ch: chalcone
CNS: central nervous system
Fl: flavanone
FRET: fluorescence resonance energy transfer
MFCs: multifunctional compounds
NPs: natural products
NFT: neurofibrillary tangles
ROS: reactive oxygen species
SPs: senile plaques
